# Computerized image understanding system for reliable estimation of spinal curvature in idiopathic scoliosis

**DOI:** 10.1038/s41598-021-86436-3

**Published:** 2021-03-30

**Authors:** Ananthakrishna Thalengala, Shyamasunder N. Bhat, H. Anitha

**Affiliations:** 1 Department of Electronics and Communication Engineering, Manipal Institute of Technology (MIT), Manipal Academy of Higher Education (MAHE), Manipal, 576104 India; 2 Department of Orthopaedics, Kasturba Medical College (KMC), Manipal Academy of Higher Education (MAHE), Manipal, 576104 India

**Keywords:** Bone imaging, Biomedical engineering, Mathematics and computing

## Abstract

Analysis of scoliosis requires thorough radiographic evaluation by spinal curvature estimation to completely assess the spinal deformity. Spinal curvature estimation gives orthopaedic surgeons an idea of severity of spinal deformity for therapeutic purposes. Manual intervention has always been an issue to ensure accuracy and repeatability. Computer assisted systems are semi-automatic and is still influenced by surgeon’s expertise. Spinal curvature estimation completely relies on accurate identification of required end vertebrae like superior end-vertebra, inferior end-vertebra and apical vertebra. In the present work, automatic extraction of spinal information central sacral line and medial axis by computerized image understanding system has been proposed. The inter-observer variability in the anatomical landmark identification is quantified using Kappa statistic. The resultant Kappa value computed between proposed algorithm and observer lies in the range 0.7 and 0.9, which shows good accuracy. Identification of the required end vertebra is automated by the extracted spinal information. Difference in inter and intra-observer variability for the state of the art computer assisted and proposed system are quantified in terms of mean absolute difference for the various types (Type-I, Type-II, Type-III, Type-IV, and Type-V) of scoliosis.

## Introduction

The three dimensional (3D) deformity of human spinal cord is referred to as scoliosis. The scoliosis is mainly characterized by the lateral curvature, which is also called as spinal curvature^1^. Scoliosis is pre-dominantly observed in teen age children. The causes for scoliosis can be conditions such as congenital, cerebral palsy and muscular dystrophy, however the cause for most scoliosis is not known.

The human spinal cord consists of 33 vertebrae, which are subdivided into five different parts namely, cervical, thoracic, lumbar, sacrum, and coccyx. The scoliosis mainly affects thoracic and lumbar regions consisting of 17 vertebrae (12 in thoracic region and 5 in lumbar region). The spine in these regions, curves abnormally towards left or right side with sideways curve of the spine more than 10 degrees being considered as scoliosis. The spinal curvature ranging between 10 to 20 degrees is considered to be mild scoliosis, between 20 to 40 degrees is considered to be moderate and above 40 degree is severe scoliosis. The majority of scoliosis cases may require follow-up and watching for worsening of the curve. Certain cases may require more aggressive treatment which could include surgery. Orthopaedic surgeons are trained for the evaluation and treatment of scoliosis. The major symptoms observed in scoliosis may include pain in the shoulders, osteoarthritis, or respiratory and cardiac problems in severe cases.

**Figure 1:**
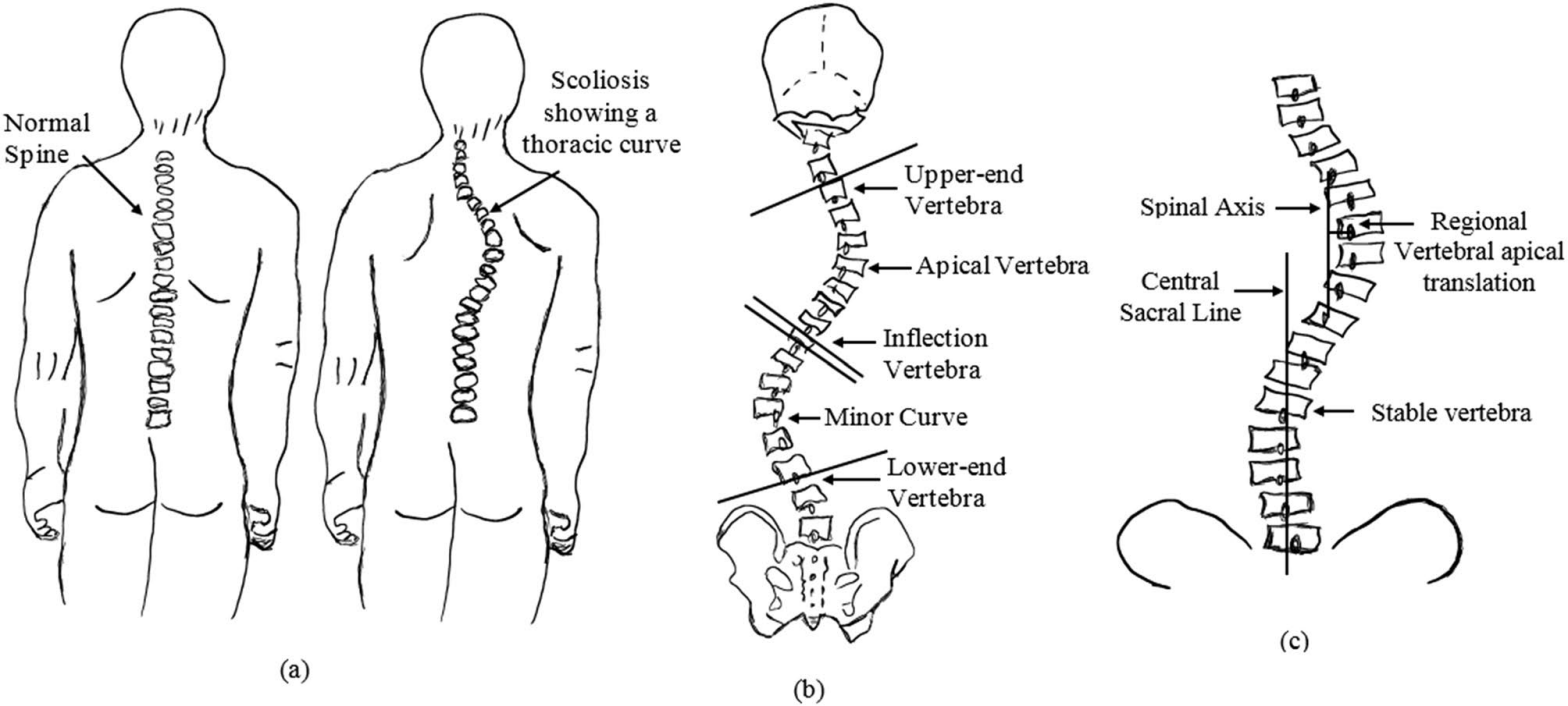
Scoliosis views: (**a**) normal versus scoliotic spine. (**b**) Definition of required anatomical landmarks. (**c**) Definition: medial axis (MA), central sacral line (CSL)^7^.

**Figure 2:**
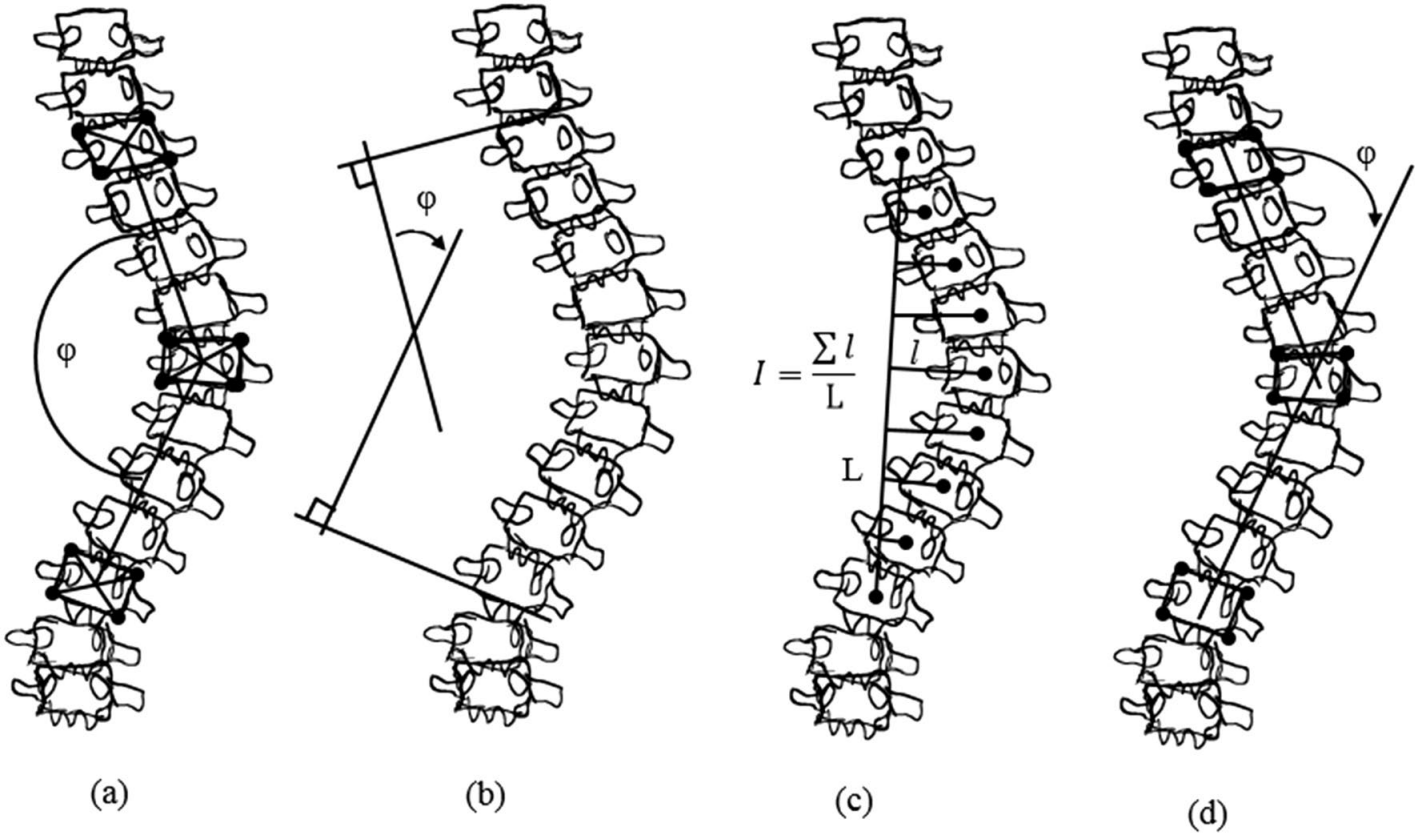
Spinal curvature evaluation (**a**) Ferguson, (**b**) Cobb, (**c**) Greenspan Index and (**d**) Diab method^8^.

The degree of spinal curvature is the key measure used by the physician in the diagnosis of scoliosis. Both two dimensional (2D) and three dimensional (3D) imaging modalities such as X-rays, CT-scans, and MRIs are used for the spinal curvature evaluation. The 2D imaging based on X-rays has advantage over other imaging techniques is that it require less capturing time, less radiation effects and is cost effective. Also, radiography images have been extensively used and proved to be successful in the analysis and evaluation of 3D deformities of scoliosis. Another advantage of radiography is that its ability capture the entire spine in the standing position. Standing radiography images are more reliable for the accurate measurement of the spinal deformity. There are several methods for the measurement of internal curvature of the spine trunk, which include Cobb, Diab, Centroid, Greenspan, and Ferguson methods (Figs. 1, 2). Among the others, Cobb method is widely used and well accepted standard for scoliosis diagnosis and treatment decisions. However, the manual measurement of Cobb angle shows poor accuracy due to the large anatomical variation of patients from different age group and the low tissue contrast of X-ray spinal image^2^. This gives raise to undesired inter-observer and intra-observer variability. So, building an accurate computer based automated measurement is essential and it is challenging for the reliable and robust assessment of scoliosis^3, 4^.

Cobb method estimates the spinal curvature by drawing a straight line which is tangent to the upper end plate of superior end-vertebra and lower end plate of the inferior end-vertebra (refer Fig. 2b). The Cobb angle is the intersection of these lines, which is accepted as the gold standard for the evaluation of curves in anterior–posterior (AP) radiographs. Diab method relies on four vertebral body corners of superior, inferior and apical vertebrae (refer Fig. 2d). By identifying centers of the required end vertebrae, estimated by intersecting the lines orthogonal to the upper end-plate and lower end plate of superior and inferior vertebrae respectively. The angle of deformity is defined by connecting center of the apical vertebrae with the end vertebrae by the intersecting lines. The vertebral body definition in centraiod method is done by connecting end vertebral body corners for the angle measurement. In continuation, the spinal curvature estimation is by drawing a tangential line on the upper and lower end-plate of superior and inferior respectively. Greenspan index method measures the deformity by identifying the centers of the end vertebrae forming the spinal line which is orthogonal to the center of each vertebra as shown in Fig. 2c. In Ferguson method, angle between the two straight lines that connect the centers of the end vertebrae with the center of the apical vertebra is taken into account for the assessment as shown in Fig. 2a. Radiography imaging technique involves identifying the end vertebrae of spinal curve and this in-turn decides the accuracy of curvature measurement. The magnitude of spinal curvature measurement needs to be precise and reproducible because it is the major factor in the clinical decision making process. The error results from variations in both radiograph production and measurement. Because of the complexity of the vertebral bodies, there are too many noises or artifacts to successfully detect the end-plate. Because of the advances in medical technology digital radiographic analysis is most popular and affordable, in terms of reliable measurement in crucial cases of idiopathic scoliosis. Automatic selection of end-vertebrae is still unsolved problem in computerized method which leads to inter and intra-observer variability^5, 6^. This work proposes automatic extraction of anatomical features to interpret required end vertebral definition for the computer assistant algorithm in terms of medial axis and central sacral line for the curvature estimation. The proposed algorithm for the extraction of CSL and MA are equally applicable to King’s as well as Lenke classifications.

## Related works

The accuracy and repeatability of spinal curvature measurement depends mainly on the operators experience, quality of image and judgment. Therefore the computer aided system are less sensitive to observer skill levels, are needed in the scoliosis assessment to improve the reliability. In computer aided system the user select end vertebra by clicking the mouse at the vertebra. The digitized computer system uses predefined vertebrae to avoid potential source of error. Finally the system can help orthopedic surgeons assess scoliosis more reliable. The reliability of digital radiographic measurement will have increasing importance as digital radiographic reading become more prevalent. Early studies on computerized measurement have reported a potential decrease in measurement error relative to manual measurement attributed to eliminating the variability of different manual protractors, the inaccuracy of manual protractors and the use of wide diameter radiographic markers.

In 1990^1^ quantitative intrinsic error in measurement procedure where introduced by Morrissy, their measurement’s is on six separate occasions. In Phase-I measurement each observer is asked to select end vertebra. In Phase-II, end vertebrae were pre-selected and constant. Phase-I is 5*°* variation and Phase-II is 3*.*8*°* for the same protractor.

Chockalingam et al.^9^, introduced the measurement with high resolution and accuracy, divided the vertebral column as a line that that can be sub dived into number of segments. State of the art computerized system by John Chung et al.^10^, are bounded by human error in-terms of end-vertebrae decision or the lines along the end vertebral plates.

In 2007, pre-selection of end-vertebrae were introduced by Brian^11^. This study includes computer measurement of digitally acquired radiographs, manual measurement of hard copy and manual measurement of traditional films. Proposed computer measurements are allowed to vary the brightness, contrast and magnifications factor.

Hitesh et al. proposed reliable assessment of Cobb angle for juvenile and adolescent idiopathic scoliosis in 2009^12^. This study measures Cobb angle on computer based digital radiograph in the inter-observer mode with predefined level of upper and lower end-plates are supplied to the observer (two observers). This study concludes reliable assessment of Cobb angle measurement by the pre-selection of upper and lower end vertebrae.

Cobb angle measurement using Active Shape Model (ASM) is proposed by Shannon et al., in 2008 for idiopathic scoliosis^13^. Set of scoliosis radiographs are used to train the software for the recognition of the vertebrae. Training part includes boundary description in terms of manual digital landmarks around the perimeter of the vertebrae.

Junhang introduced Hough transform and snake model for Cobb angle and vertebral rotation. These algorithms were integrated with shape priors to improve the performance of evaluation. Since selection of end vertebrae was a possible source of error that had no relation to the technique involved in the Cobb measurement, end vertebrae were pre-selected in this study^14^. Reliable analysis of spinal curvature using Cobb angle has been studied by Eiten et al.^15^. Their study uses Picture Archiving Computer System (PACS) for precise definition of anatomical landmarks for angle measurement which includes indices, length of joints, limbs and spine. This results in decorrelation of required bony landmarks as insignificant information.

Technical report has been submitted by^5^ to ensure that reliable measurement of Cobb angle using digital and manual, which conveys that the assessment is an important parameter in-terms of posterior–anterior radiograph. This study also encourages use of digitized radiograph for routine clinical practice. This report also illustrate the clinical advantage for appropriate assessment of spinal curvature which is an unsolved problem in end-vertebra selection.

Bidur Khanal^16^ introduced a novel framework to detects vertebrae as objects, that estimates the 4 landmark corners of each vertebra separately. Their approach was promising because they are predicting the vertebrae before the landmarks. However, cropping all test images will not generalize as more robust object detector trained with images having negative samples from skull and pelvic regions.

In 2017, Ming-Huwi^17^ proposed CNN based network to reduce the influences of inconsistent intensity distribution of vertebrae in the spine in spinal curvature estimation, which includes the U-Net, the dense U-Net, and residual U-Net, to segment the vertebrae. Their study resulted with high correlation to manual assessment by clinical doctors.

In 2019, Yongcheng^18^ and his team attempt to extract the Spine contour through deep learning approach in the feature map plane. The features used in their study includes gradient magnitude and gradient histogram. Extraction of these features vary exponentially in the scoliosis pattern, which misleads the decision process. As idiopathic scoliosis is a structural deformity, its deformity is clearly visible in the anterior- posterior view, as it is directly exposed to radiation plane.

Abdullah-Al-Zubaer Imran and his team in 2020^19^, performed vertebrae segmentation and labelling using progressive U-net. They imposed minimum size on the number of contour pixels. Proposed progressive U-net illustrates the Cobb angle estimation.

## Materials and methods

This study was conducted in collaboration with department of Orthopaedics, Kasturba Medical College, Manipal, completed in 2015. The experiments were performed in accordance with the hospital guidelines. This study involves anonymized antero-posterior radiographs of adolescent idiopathic scoliosis. This study was approved by Departmental Review Committee, Department of Orthopaedics, Kasturba Medical College, Manipal, Manipal Academy of Higher Education (MAHE). This study was done before the revised guidelines for research in lndia (lCMR 2017) were released. For retrospective studies, KMC and KH Institutional Ethics Committee waivers informed consent from the patient/parents. As it was a low risk study, only Departmental Review Committee approval was sought, and it was exempted from approval by the KMC and KH Institutional Ethics Committee.

Structural deformity of spinal column in the lateral direction leads to scoliosis disorder. The displacement of the spinal column from the original position to the new position has been evaluated in terms of medial axis and central sacral line. The medial axis or spinal mid-line (MA) is defined as the curve passing through the centroids of the vertebral bodies (Refer Fig. 1c). Central sacral line (CSL) is a vertical line representing the global axis, and it is drawn from the center of the cervical-1 (C1) vertebra to sacrum-1 vertebra (S1) as shown in Fig. 1c. Spine with scoliotic disorder in which MA are enforced in the direction of deformity. The CSL is unaffected because scoliosis is defined from thoracic to lumbar region but the CSL definition starts from cervical to sacrum region. The displacement of the MA from its original position to the new position are good enough to define the required anatomical land marks like (SEV, IEV and AP) for spinal curvature estimation using Ferguson, Cobb, Greenspan and Diab^20^.

### Materials

Anterior Posterior (AP) radiographs of 250 patients with idiopathic scoliosis are used for the study obtained from the department of Orthopaedics, Kasturba Medical College, Manipal, Manipal Academy of Higher Education (MAHE), Karnataka, India. These radiographs are taken in a conventional standing posture at a fixed distance of 228 cm from X-ray source. These AP radiographs includes thoracic, thoracic lumbar and lumbar scoliosis with all five different types of scoliosis. Radiographic images of 256 gray levels with size of 925 pixel heights by 475 pixels wide are used.

Two hundred and fifty cases of adolescence idiopathic scoliosis are considered in the present study. The cases considered are recorded in the first visits and the study is performed over a period of 3 years. Congenital and neuromuscular spinal deformities are excluded from the study.

### Extraction of MA and CSL

**Figure 3:**
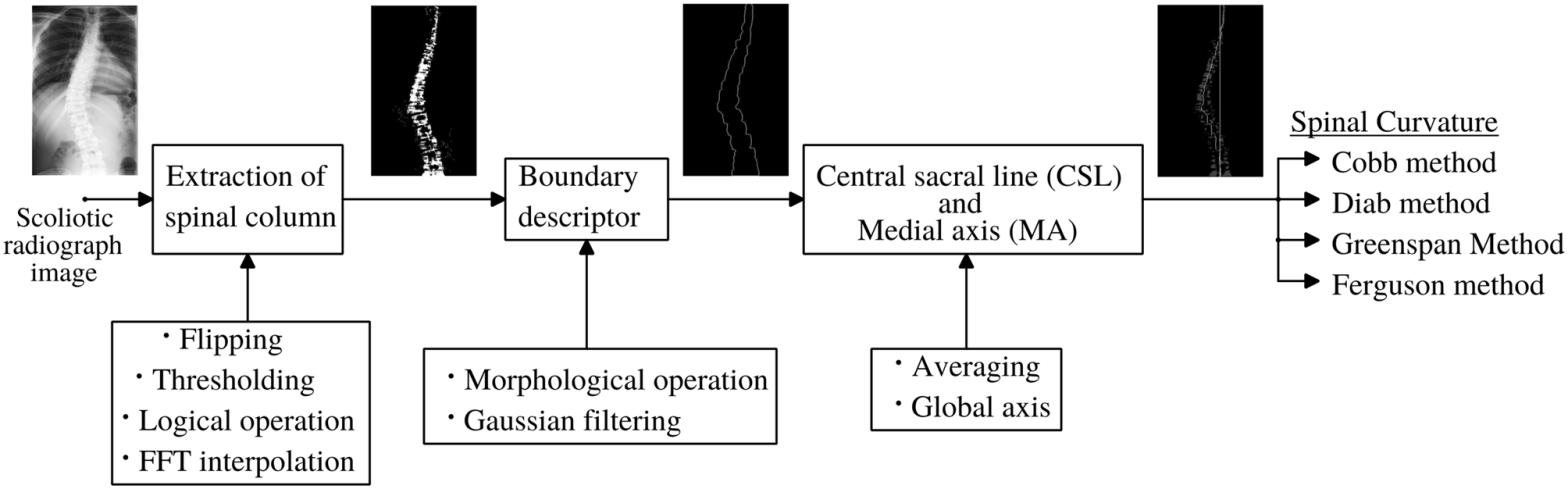
Spinal curvature evaluation.

Manual definition of CSL and MA are time consuming and imprecise. The point to point correspondence between CSL and MA defines required superior, inferior and apical vertebrae. Proposed methodology extract the CSL and MA for the automatic identification of the vertebral parameters for the different spinal curvature estimation techniques as shown in Fig. 3.

#### Extraction of spinal column

Optimum global threshold removes all the other pixels with higher intensity in the pre-processing step. The pre-processed image will be a binary image with white pixel region enveloping a minimal extraneous content. Logical operators are applied to retrieve back the original X-ray features localized to the enveloping region. To partially suppress the unnecessary side information, interpolation has been proposed in terms of fast Fourier transform (FFT) method. And this FFT method adds interpolated data to the original data as shown in algorithm1.

Algorithm 1 Extraction of Spinal Column
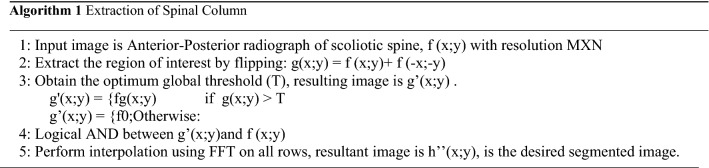


#### Boundary description

Erosion operation has been performed on the resultant image after the extraction of the spinal column. Then subtract the eroded image to result into two boundaries, out of which large boundary had been chosen. Boundary operation is done with Gaussian filter and finally convert them into binary image. This algorithm is summarized and is as shown in Algorithm 2. As digital technology continues to advance and become increasingly affordable, digital radiograph analysis will become common place. Contrast, sharpness and visualization details may be manipulated and enhanced with digital techniques. However the content of the radiographic film cannot be improved and landmark identification may persist.
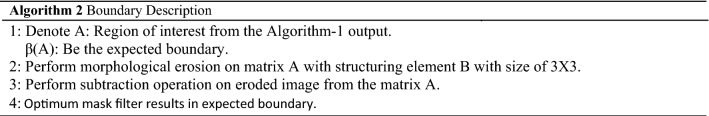


#### Definition of CSL and MA

The boundary representation consists of pair of co-ordinates. For every co-ordinate there exist two X-values. The average of these two X-vales will be the X-coordinates of the medial axis. As discussed in the introduction, definition of the required end vertebrae identification is based on the deformity of the spinal column, it has been represented by MA. Quantification of MA deformity is carried out with reference to the data from its CSL. Point to point displacement between MA and CSL represents the deformity. Developed computerized algorithm reads the coordinates of CSL and MA. Computer assisted system will identify the indication of the curvature by checking the X-coordinate where exactly is it different than X-coordinate of the CSL.

Similarly, the curvature endings are identified by checking once again with overlapping of X-coordinate between CSL and MA. The maximal displacement between X-coordinate of CSL and MA represents the apical vertebra.

### Spinal curvature estimation

The performance of spinal curvature measurement depends on the accurate identification of anatomical landmarks. The anatomical features are automatically located by using CSL and MA algorithms. Further, the spinal curvature estimation from the obtained landmarks is done by using different methods such as Ferguson, Cobb, Greenspan and Diab. The proposed inference in terms of anatomical description for spinal curvature estimation is given in Table 1.

**Table 1:** Proposed inference in terms of anatomical description for spinal curvature estimation.

Method	Required landmarks anatomical	Anatomical description	Outcome of this work
Ferguson method	(a) Line joining from SEV to AV	(a) By knowing the x-co-ordinate of MA, its intersection with CSL will identify SEV	
	(b) Line joining from AV to TEV	(b) Maximum displacement between x-co-ordinates of CSL and MA represents the apical vertebrae	
Cobb method	Identification of SEV and IEV	(a) Proposed system will identify the indication of the curvature by checking the x-co-ordinates where exactly is it different than x- co-ordinates of the CSL	
		(b) Similarly the curvature endings are identified by checking once again with overlapping of x coordinate between CSL and MA. The maximal displacement between the x-co-ordinates between CSL and MA represents the apical vertebrae	
Greenspan Index	(a) Identification of starting and ending vertebrae	(a) Starting and End vertebrae are identified by the inter-secting point between CSL and MA	
	(b) Displacement of each centroid to its CSL	(b) Difference in the x-co-ordinate point of all MA-axis defines this method	
Diab	(a) Centroid of three successive starting vertebrae	(a) Upper intersecting point between MA and CSL taken as mid vertebrae	
	(b) Centroid of three successive ending vertebrae of the curve	(b) Similarly lower-intersecting point between MA and CSL taken as mid-point for end vertebrae	

### Ethical approval

Manipal Academy of Higher Education (MAHE) encourages faculty members to publish their research outcome in quality journals through "Incentive for research publication" program. The corresponding and co-authors of this article are under the functional body of this program.

## Results and discussion

The 250 adolescent scoliotic radiographs collected from the associated hospital have been grouped equally. Different observers are assigned to evaluate the spinal curvature using manual, computer assisted and proposed methods. Each observer is provided with all types of radiographs. In case of manual measurement, each observer is informed to identify the required end vertebrae for the best of their knowledge and experience. The experts would calculate the spinal curvature using ‘ruler and pencil’ procedure. In computerized method, identification of required anatomical landmarks (marking of superior and inferior end vertebrae) are done by the each observer independently. Estimate the spinal curvature based on the identified anatomical landmarks with the computer assistance. This computer assisted method eliminates the observer error introduced by the ‘ruler and pencil procedure’. In the proposed method, it identifies the required end vertebrae automatically. After the automated identification, observer is asked to identify the corners of the end vertebrae. This in-turn may introduce minimal observer error. Successive procedures will be taken care by computer assistance method.

### Experimental results

Consistency, or agreement among the Surgeons (individuals) arises due to the variability among human observers in the decision of superior end vertebra (SEV), inferior end vertebra (IEV) and apical vertebra (AV). In our study, agreement among the surgeons in-terms of SEV, IEV and AV definition is referred as inter-rater variability which is assessed using kappa statistic. This study includes two Junior resident who are trained for this work are adopted for evaluation. Cohen’s kappa statistic check the reliability among the vertebral labeling. The observed agreement is defined as percentage of correct labeling of anatomical landmarks (SEV, IEV and AV). The expected agreement is based on how much agreement would be expected to be present by chance alone in the localizing the vertebrae. Each study has been carried out with 50 adolescent idiopathic radiographs. Table 2 shows the interrater variability in terms of kappa statistic. It is clearly observed from the kappa statistic that the proposed method shows a good agreement.

**Table 2:** Kappa statistic for inter-rater reliability assessment.

Anatomical landmarks	Manual method			Proposed method		
	*Kappa-a*			*Kappa-b*		
Study group	Study-1	Study-2	Study-3	Study-1	Study-2	Study-3
SEV	0.542	0.48	0.56	0.75	0.84	0.81
IEV	0.621	0.57	0.79	0.8	0.83	0.9
AV	0.59	0.58	0.49	0.91	0.88	0.85

*Kappa-a* Interrater variability between observer-1 and observer-2 on pain radiagraph.

*Kappa-b* Interrater variability between observer-1 and observer-2 on CSA and MA extracted radiograph.

### Experimental analysis

**Figure 4:**
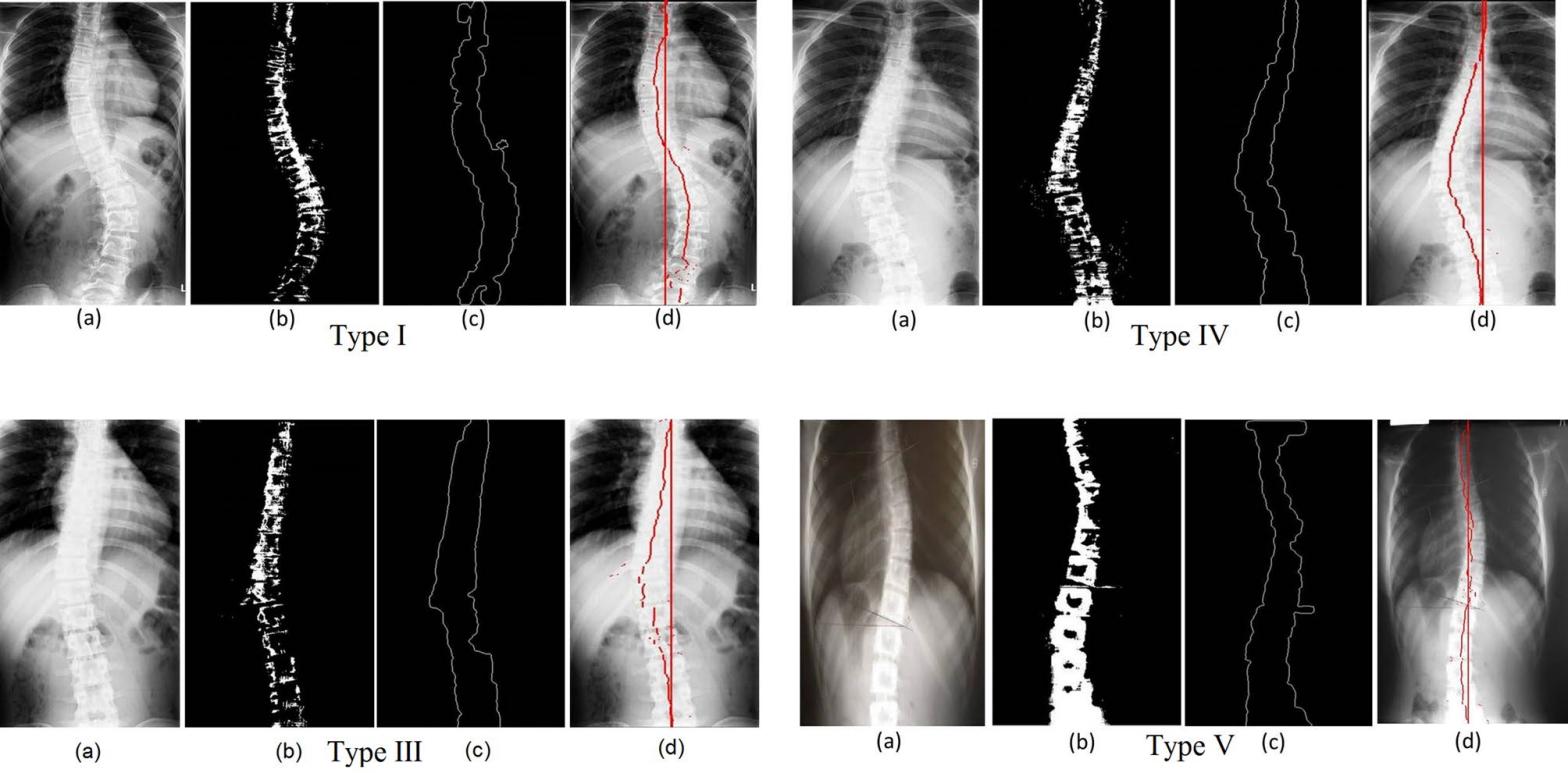
Images of Type-I, Type-III, Type-IV and Type-V for (**a**) input radiograph, (**b**) segmented vertebral column, (**c**) extracted boundary and (**d**) automated definition of MA and CSL.

We have grouped the given radiographs into different classes based on the type of deformities, which includes all types of scoliosis. The resultant spinal columns are represented in Fig. 4-Type I-b, Type III-b, Type VI-b and Type V-b. Determination of spine axis needs only the boundary coordinates, these boundary coordinates are retained using boundary descriptors as shown in Fig. 4-Type I-c, Type III-c, Type VI-c and Type V-c. Coordinates of the MA are the average coordinates of the boundary in X- axis and CSL is vertical line drawn from the centroid of C1 to S1. Figure 4-Type I-d, Type III-d, Type VI-d and Type V-d illustrates the same.

**Figure 5:**
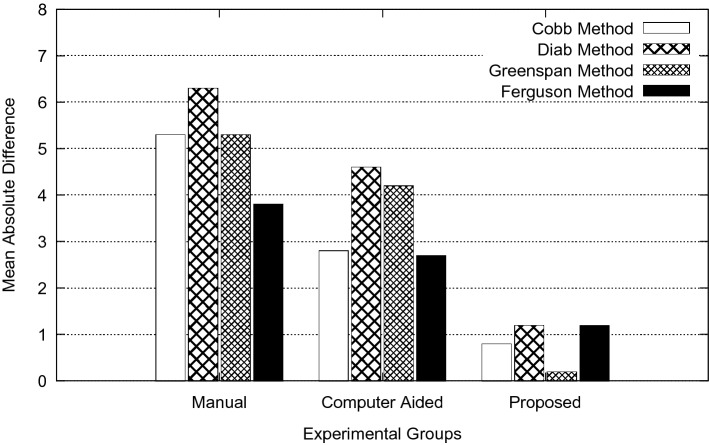
Type I (a) mean absolute difference (MAD) in inter observer variation.

**Figure 6:**
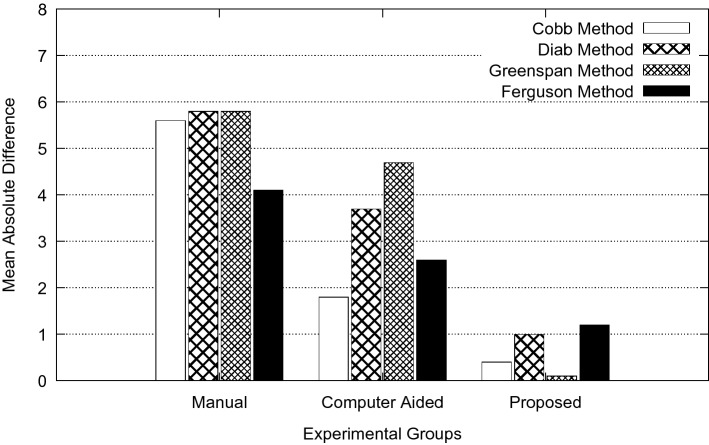
Type II (a) mean absolute difference (MAD) in inter observer variation.

**Figure 7:**
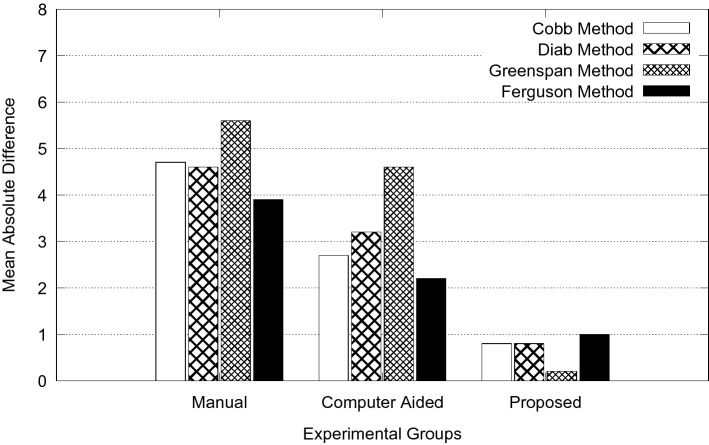
Type III (a) mean absolute difference (MAD) in inter observer variation.

**Figure 8:**
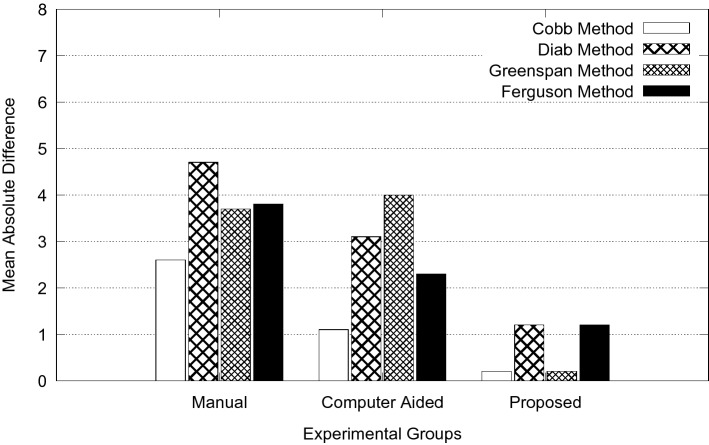
Type IV (a) mean absolute difference (MAD) in inter observer variation.

**Figure 9:**
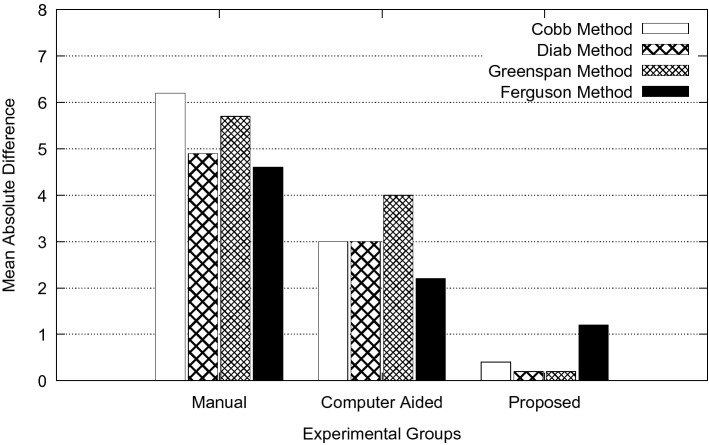
Type V (a) mean absolute difference (MAD) in inter observer variation.

The overall goal of this study is to reduce measurement error in spinal curvature estimation with different technique such as Cobb estimation, Ferguson method, Centroid method and Diab procedure. The mean absolute difference in terms of inter-observer error for manual, computer assisted and proposed methods are presented using bar charts as shown in Figs. 5, 6, 7, 8 and 9. Routine clinical method with manual assessment causes error in different stages, while deciding the superior and inferior end vertebrae and its upper and lower end plate detection. In the low-dose radiograph results with cumulative effect while drawing line across the end-plate because of wrong selection of end-vertebrae. Computer assisted system will eliminate the error introduced in the line joining on the end-plates and as well as on connecting all the centroid points from starting point of the deformity to end point.

To identify the severity of the curve, it is necessary to evaluate the inclination of the vertebral bodies. The limiting vertebrae are the most oblique one while the apical vertebra is the most rotated and wedged one. The possible errors introduced in the spinal curvature measurements are due to the identification of the required anatomical landmarks. It is difficult to draw the lines tangent to the vertebral bodies because of vertebral deformity. To reduce variability in spinal curvature measurement for the scoliosis assessment, a computerized method was developed. Proposed method automatically measures the spinal curvature on posterior-anterior radiographs. The brightness and the contrast were adjusted on the radiograph for the proper selection of top and bottom vertebrae. Continuation of this results in manual decision on the spinal curvature estimation. The end-vertebral tilt is mostly towards the concavity of the spinal column for the measurement of spinal curvature. However, the manual measurement of spinal curvature depends on experience and judgment. This results in errors due to selecting different end vertebrae and estimating different slopes of the vertebrae. In addition, the manual measurement is tedious and time-consuming. This proposed work is a new approach which extracted the MA and CSL using advanced computerized image understanding system for the decision of the required anatomical landmarks. The participants involved in testing are orthopedic surgeons, who had several years of experience. Manual marking of end vertebrae were marked for measurement with soft lead pencil and after measurement had been recorded the marking were erased with trichloracetone. Each observer took approximately 5 min to identify required end vertebrae and to estimate the spinal curvature with ruler and pencil. Each examiner recorded his choice for different groups of scoliosis radiographs. This experimental study involves different spinal curvature estimation (Cobb Method, Ferguson method, Diab method and Greenspain method). We have grouped each type of scoliosis into five different categories as categorized by King’s classification. Each group is defined with minimum 10 scoliotic radiograph. We assigned three participants (experienced) for the estimation process. All their measurements are tabulated for all types for every radiograph in the group. Each person comes with 30 different reading within the group (10 set of readings for manual, computerized and proposed). The absolute difference between these reading in each set is considered for the measurement statistics.

### Discussion and scope

From the result plots (refer result plots in Figs. 5, 6, 7, 8, 9), we can observe that the observer error variation is more in case of Greenspain and Diab methods. This is because of more manual intervention in-terms of identification of centroid of all the vertebrae starting from the curvature to the end of the curvature along with the displacement of the centroid point from the CSL point. Computer assisted system will have better estimation, but still this method also faces all the above mentioned anatomical identification task. But proposed method infers very good reliability in terms of estimation with minimum difference for inter observer evaluation. Proposed method doesn’t need to identify any of the centroid pints which eliminates major human error, because of automatic definition of CSL and MA, which automatically defines all the required vertebrae along with its centroids. This inference has been reflected in the results in terms of minimum error. In comparison to other two estimation methods like Cobb and Ferguson method, the manual intervention is minimal, which includes identifying the superior, inferior and apical vertebrae. From the table we can observe that, inter observer error introduced is less in compare to other methods. When we observe the error in terms of different types of scoliosis curvature as defined by King’s, Type-1. Type-2 and Type 5 are more prone to error because of confusion in the identification of superior and inferior vertebrae. In these estimation technique, starting and end curvature regions are extended, which leads to more confusion in the vertebrae identification. But in Type-3 and Type-4 have better results because of the curvature nature in selecting the required end vertebrae for the angle estimation.

## Conclusion

Spinal curvature measurement needs identification of the end vertebrae. Human intervention is mandatory for deciding the end vertebral landmarks. Due to human decision at different levels inter and intra observer errors are introduced. Computer assisted systems are useful to eliminate these errors up to some extent. It works on digital reconstruction of manually identified landmarks. Current computerized system evaluates the landmarks based on the enhanced or edge detected radiographs. The proposed computerized image understanding system completely automates the quantification of the spinal curvature by the automatic definition of end vertebrae using extracted CSL and MA. These automatic extracted CSL and MA can be further extended to the estimation of thoracic kyphosis and lumbar lordosis in standard lateral radiographs, where there is no vertebral anomalies.
